# Extracellular vesicles for cancer therapy: potential, progress, and clinical challenges

**DOI:** 10.3389/fbioe.2024.1476737

**Published:** 2024-09-27

**Authors:** Lili Ren, Dingmei Zhang, Long Pang, Shiyu Liu

**Affiliations:** ^1^ State Key Laboratory of Oral and Maxillofacial Reconstruction and Regeneration, National Clinical Research Center for Oral Diseases, Shaanxi Key Laboratory of Stomatology, Department of Oral Biology and Clinic of Oral Rare Diseases and Genetic Disease, School of Stomatology, The Fourth Military Medical University, Xi’an, China; ^2^ State Key Laboratory of Oral and Maxillofacial Reconstruction and Regeneration, National Clinical Research Center for Oral Diseases, Shaanxi International Joint Research Center for Oral Diseases, Center for Tissue Engineering, School of Stomatology, The Fourth Military Medical University, Xi’an, China; ^3^ Department of Orthopaedic Surgery, Affiliated Hospital of Zunyi Medical University, Zunyi, China; ^4^ College of Basic Medical Science, The Shaanxi Key Laboratory of Brain Disorders, Xi’an Medical University, Xi’an, China

**Keywords:** extracellular vesicles, EV analogs, origins, purification, drug delivery, cancer treatment

## Abstract

Extracellular vesicles (EVs) play an important role in normal life activities and disease treatment. In recent years, there have been abundant relevant studies focusing on EVs for cancer therapy and showing good performance on tumor inhibition. To enhance the effectiveness of EVs, EV analogs have been developed. This review summarizes the classification, origin, production, purification, modification, drug loading and cancer treatment applications of EVs and their analogs. Also, the characteristics of technologies involved are analyzed, which provides the basis for the development and application of biogenic vesicle-based drug delivery platform for cancer therapy. Meanwhile, challenges in translating these vesicles into clinic, such as limited sources, lack of production standards, and insufficient targeting and effectiveness are discussed. With ongoing exploration and clinical studies, EV-based drugs will make great contributions to cancer therapy.

## 1 Introduction

Cancer is a significant major global concern with about 20 million new cases and 9.7 million deaths in 2022 (Bray et al., 2024). Demographics predict the number of new cases will reach 35 million by 2050. The cancer burden has grown over time worldwide due to complex reasons, including population expanding and aging, increasingly shifting from older to middle aged, and changes in the prevalence of associated risk factors (Cao et al., 2021; Siegel et al., 2024). Despite the importance of cancer drugs, many challenges remain. Chemotherapy drugs are commonly used in clinical practice for various cancers, but face challenges like lack of specificity, short half-life, and multi-drug resistance (Gong et al., 2021; Mellman et al., 2011). Immunotherapy, including immune checkpoint inhibitors, is a significant advancement for cancer therapy, with 14 PD-1/PD-L1 antibodies approved globally in 2021 (Galon and Bruni, 2020; Yin et al., 2021; Poggio et al., 2019; Li et al., 2022; Upadhaya et al., 2021). However, these inhibitors cannot fully restore anti-tumor immunity, leading to potential resistance and treatment failure (Lesterhuis et al., 2011). With the development of nanotechnology, nanomaterials as drug carrier for cancer therapy may reduce the shortcomings above.

Nano-drug delivery systems, such as liposomes (Ren et al., 2016; Guimarães et al., 2021), polymeric nanoparticles (Bao et al., 2021; Ghosh and Biswas, 2021), dendrimers (Lim et al., 2013; Tarach and Janaszewska, 2021), nanofibrous and scaffolds (Zhang X. et al., 2021; Han R. et al., 2023), carbon nanoparticles (Burnett et al., 2020; Sahu et al., 2020), mesoporous silicon (Nguyen et al., 2019; Xu et al., 2024), quantum dots (Dubertret et al., 2002; Liu et al., 2020), and metallic and magnetic particles (Ren et al., 2023; Attarilar et al., 2020), can deliver drugs to tumor cells and diseased regions via encapsulation, attachment, conjugation, or charge adsorption. Passive targeting leverages the enhanced permeability and retention (ERP) effect. While active targeting uses surface modifications like antibodies and aptamers to reduce toxicity in normal cells, increase half-life and solubility and anti-tumor effects of drugs. In addition to acting on tumor cells, nano-drug delivery systems can also target to tumor microenvironment and immune system to treat cancer (Cheng et al., 2021; Izci et al., 2021). Despite extensive research over the past 2 decades, few nano-drugs, like liposomes and certain nanoparticles, have reached clinical use (Bobo et al., 2016; Raj et al., 2021). Challenges include potential toxicity, immune responses, and differing effects in human organs compared to cell and animal models, complicating clinical translation (Tenchov et al., 2022). A safe and effective treatment platform is needed.

Unlike traditional nanoparticles, extracellular vesicles (EVs) are biogenic lipid bilayer particles with low immunogenicity and high biocompatibility, making them suitable for cancer therapy. EVs, including exosomes (about 30–150 nm in diameter), microvesicles (100 nm-1 μm) and apoptotic bodies (50 nm-5 μm), are rich in proteins, lipids, and cytoplasmic components, involved in various physiological and pathological processes (Yong et al., 2022; Doyle and Wang, 2019; Guo et al., 2024). Certain EVs, like those from immune cells, retain the functions of their parent cells and can work on tumor process directly (Shin et al., 2022; Hong et al., 2021). EVs as drug delivery systems can merge with recipient cell membranes, avoid lysosomal phagocytosis, and deliver drugs directly into the cytoplasm, enhancing drug efficiency (Gui et al., 2024; Herrmann et al., 2021). Additionally, EV-mediated drug delivery boosts anti-tumor immune responses (Yong et al., 2022; Tian et al., 2023), and is effective against multi-drug resistant tumors (Zhang H. et al., 2021), making it a promising tumor treatment strategy.

**FIGURE 1:**
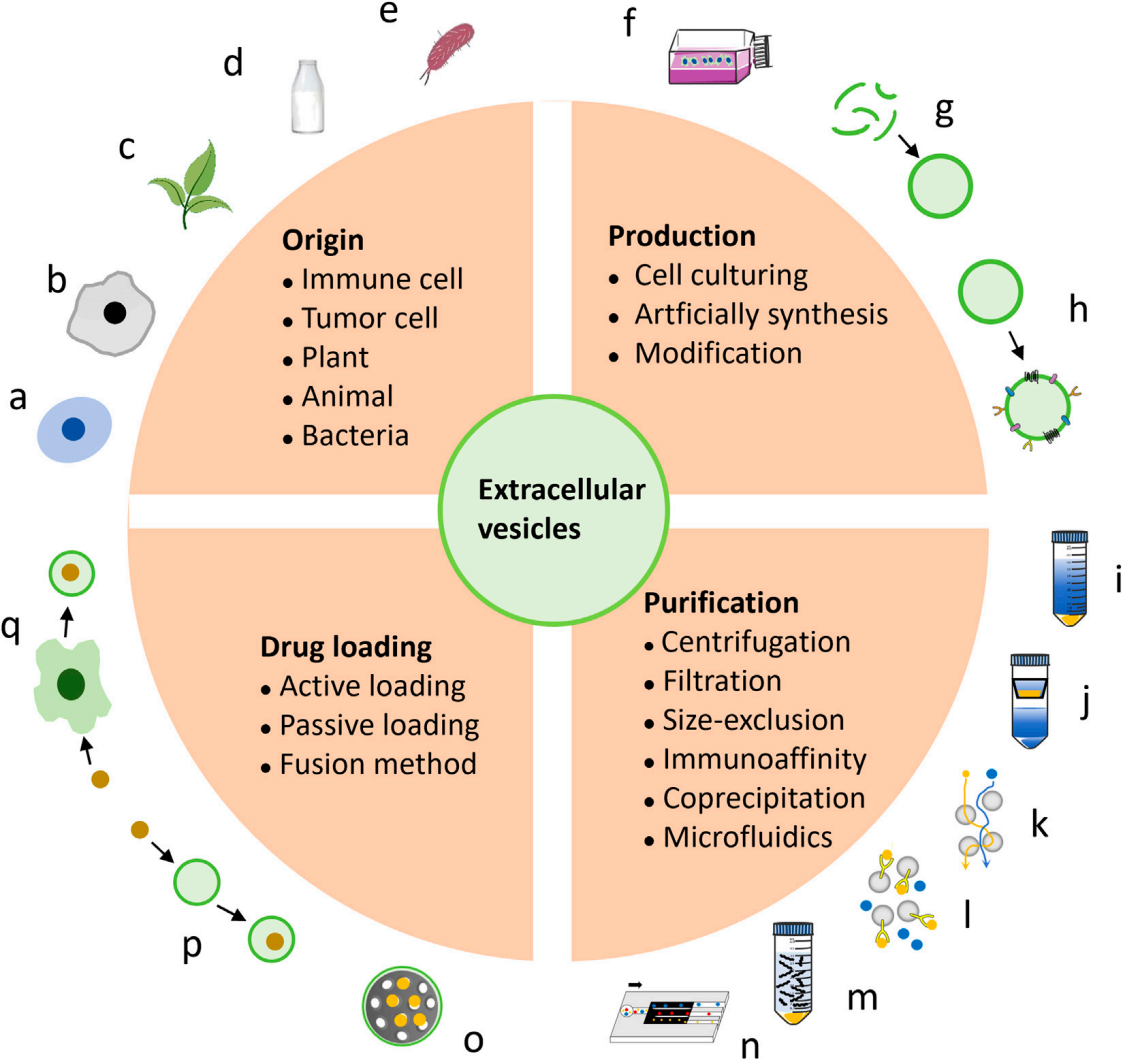
The introduction encompasses the origin, production, and construction of EVs as platforms for cancer therapy. EVs can be derived from various sources, including immune cells **(A)**, tumor cells **(B)**, plants **(C)**, bovine milk **(D)**, and bacteria **(E)**. The main approaches for producing EVs involve cell culturing **(F)** and artificial synthesis **(G)**, with further modifications **(H)** enhancing their efficacy. EVs purification techniques include centrifugation **(I)**, filtration **(J)**, size-exclusion chromatography **(K)**, immunoaffinity **(L)**, coprecipitation **(M)**, and microfluidics **(N)**. The incorporation of anti-cancer drugs into EVs can be achieved by active **(O)** or passive **(P)** loading, as well as fusion methods **(Q)**.

Exosomes, the smallest and most functional EVs, are particularly prominent in cancer therapy research (Yong et al., 2020). This review primarily focuses on exosomes among EVs. Notably, EV analogs reassembled with cell membranes share structural similarities with EVs and offer versatile drug delivery options, which are also discussed here. Further optimization is needed to enhance EV properties and applications, such as surface modification, vesicles fusion, and integration with other nano-systems. Leveraging the natural advantages of vesicles and the customizable nature of nanotechnology, engineered biogenic vesicles are advancing rapidly. The classification of EVs from various sources was detailed, along with methods for their production, purification, and drug loading for cancer therapy (Figure 1).

## 2 Extracellular vesicles from different origins

Most cells, including immune cells, stem cells, tumor cells and somatic cells, are capable of secreting EVs, which are detectable in various body fluids such as blood, saliva, urine, and amniotic fluid. However, the limited availability of these body fluids poses challenges for large-scale production and scalability. Cell lines have been commercialized and present a viable option for mass production of EVs. Furthermore, plant-derived nanovesicles, milk-derived exosomes, and bacterial EVs have demonstrated potential in tumor treatment and are suitable for large-scale production. The primary sources and characteristics of EVs have been summed up here (Table 1).

**TABLE 1:** EVs from different origins.

Origin	Advantage	Disadvantage	Administration	Application	Ref
Immune cell: T cell, macrophage, NK cell, DC cell	Potential immune effect, well-characterized, modifiable	Limited source, high cost	Intravenous injection (i.v.)	Inhibit tumor cell proliferation, improve immunity function, improve the effect of chemotherapeutics	Shin et al. (2022), Hong et al. (2021), Matchett and Kornbluth (2023)
Tumor cell: breast/lung/liver cancer cell	Potential tumor targeting effect, modifiable	High cost, potential toxic side effect	i.v., intrapleural infusion	Induce tumor cell apoptosis, improve immunity function	Jiao et al. (2022), Bose et al. (2018), Guo et al. (2019)
Plant cell: tea leaf, Chinese traditional medicine, grapefruit, purple cabbage	Safe, rich source, potential drug function	Immature technology, low yield and purity	i.v., intraperitoneal injection (i.p.), Poros (po)	Improve immunity function, induce tumor cell apoptosis	Cao et al. (2023), Yan et al. (2024), Zhao et al. (2023)
Animal cell: bovine milk	Safe, rich source, oral bioavailability	Immature technology, potential health risks	po, i.v	Improve immunity function, inhibit tumor cell proliferation	Chen et al. (2024), Del Pozo-Acebo et al. (2021)
Bacterium: gram-positive bacterium, gram-negative bacterium	Rich source, modifiable	Potential toxic side effect and hypersensitivity	i.v	Improve immunity function, inhibit tumor cell proliferation	Xiao et al. (2024), Zhao et al. (2024)

### 2.1 EVs from immune cells

Immune cell-derived EVs retain the parent cells ‘characteristics, exhibit immune properties, regulate immune responses, and can deliver drugs for tumor therapy. For instance, EVs from CD4^+^T cells can inhibit tumor growth through CD8+T cell activation (Shin et al., 2022). EVs from CD8^+^ T cells with PD-1 and TGF-β receptors can block PD-L1 on cancer cells, clear TGF-β from the immunosuppressive environment, and prevent cytotoxic T cell depletion. Granzyme B directly kills tumor cells and shows strong anti-tumor effects in mice with solid tumors (Hong et al., 2021). Exosomes from M1 macrophages can repolarize M2 tumor-associated macrophages to M1, enhancing the release of pro-inflammatory cytokines and anti-tumor immunity (Choo et al., 2018). EVs from natural killer (NK) cells induce tumor cell apoptosis via highly expressed cell death receptors, and can inhibit tumor cell proliferation by reducing phosphorylated extracellular signal-regulated kinase and phosphorylated protein kinase B (Aarsund et al., 2022; Matchett and Kornbluth, 2023).

Modified immune cells EVs can boost the anti-tumor effects. For example, exosomes from HEK293T cells with PH20 hyaluronidase degrade hyaluronic acid, improving Doxorubicin (Dox) penetration and CD8^+^ T cells infiltration, thus enhancing Dox’s anticancer activity (Hong et al., 2018). iRGD-modified dendric cell (DC) exosomes effectively deliver Dox to breast cancer (Tian et al., 2014). Dox-loaded macrophage vesicles modified with biotin and folate-conjugated DSPE-PEG also significantly improve Dox’s anti-tumor efficiency (Zhang et al., 2017). A study created hybrid vesicles by fusing platelet-derived, M1 macrophage-derived, and tumor cell-derived vesicles with high SIRP-α mutant expression. These vesicles could effectively accumulate in the wound site after melanoma surgery, block SIRP-α/CD47 interactions, boost macrophage response to cancer recurrence, enhance anti-tumor T cell immunity, and reduce side effects caused by systemic perfusion (Rao et al., 2020). Therefore, immune cell-derived EVs demonstrate good drug transport abilities and corresponding immune functions. However, the availability of immune cells is limited, and the isolation process is intricate. For instance, CD4^+^ T cells were obtained from blood and stimulated by IL2 (Shin et al., 2022), while NK3.3 cells were immortalized using lentivirus encoding to overcome growth arrest (Matchett and Kornbluth, 2023). Clinical trials involving DC-derived EVs demonstrated only modest therapeutic efficacy due to various complex factors (Cao et al., 2022). Future research can focus on more rational construction of immune cells and combination therapies.

### 2.2 EVs from tumor cells

Current research aims to enhance drug targeting to tumor cells using tumor extracellular vesicles (TEVs) or tumor cell membranes as nanocarriers (Jiao et al., 2022; Yong et al., 2019; Liu C. et al., 2019). TEVs resemble tumor cells and can be recognized and absorbed by them, aiding targeted molecule delivery. For instance, breast TEVs were electroporated to release the content and load triphenylphosphine-modified P53, eliminating the toxic side effects and hypersensitivity of undesirable tumor cell components and P53. These vesicles effectively targeted breast cancer cells with improved transmembrane infiltration and cell apoptosis (Jiao et al., 2022). A study explored breast TEVs loaded with miRNA and gold-iron oxide nanoparticles, which enhanced cell adhesion and uptake, allowing targeted tumor accumulation of drugs and contrast agents for cancer treatment and nuclear magnetic imaging (Bose et al., 2018). M2pep, a peptide targeting M2 macrophages, was modified on liver cancer cell membranes coated on PLGA nanoparticles with D-lactic acid. By leveraging the homing ability of tumor cell membranes, these engineered vesicles can deliver D-lactic acid to M2 macrophages, thereby converting them into the M1 phenotype within the tumor microenvironment. This transformation enhances the efficacy of anti-CD47 antibody treatment, resulting in improved long-term survival in mouse models of liver cancer (Han S. et al., 2023). In a separate study, vesicles derived from tumor cell membranes and loaded with methotrexate were administered to 11 patients with advanced lung cancer and malignant pleural effusion. The results demonstrate that these vesicles significantly inhibited the progression of malignant pleural effusion without inducing notable toxicity. The presence of tumor cells and CD163^+^ macrophages in malignant pleural effusion was significantly diminished. Concurrently, both CD8^+^ and CD4^+^ T cells were activated to secrete immune factors. These findings indicate that this system can effectively target malignant tumor cells and modulate the immune microenvironment, presenting a promising strategy for cancer therapy (Guo et al., 2019). However, TEVs play a crucial role in shaping the tumor microenvironment, thereby facilitating tumor progression. The proteins present on TEVs have the potential to induce biological toxicity. These risks warrant significant attention. Therefore, a judicious selection of parental cells and delivery systems is imperative to maximize therapeutic efficacy while minimizing the promotion of cancer (Xu et al., 2022).

### 2.3 EVs from plant

Plant nanovesicles (PNVs), secreted by plant cells, are derived from a diverse range of sources. PNVs, characterized by their rich bioactive substances, excellent biocompatibility and stability, high permeability, and low immunogenicity and toxicity, represent an ideal natural green drug delivery platform (Cao et al., 2023). Xiao and colleagues were the first to identify natural exosome-like nanomaterials derived from tea leaves, which are rich in proteins, lipids, and bioactive small molecules. These tea-derived nanomaterials exhibit the capability to specifically target macrophages and enhance the secretion of anti-inflammatory factors by these cells, thereby providing a therapeutic effect against colon cancer with minimal side effects (Zu et al., 2021). Furthermore, this team discovered that fresh tea contains exosome-like nanovesicles abundant in pharmacologically active molecules, including lipids, proteins, catechins, and flavonoids. The nanovesicles were internalized by tumor cells, thereby stimulating the production of intracellular reactive oxygen and inducing cell apoptosis. Whether they were administered intravenously or orally, these vesicles demonstrated efficacy in controlling the proliferation of breast cancer cells (Chen et al., 2023). Furthermore, vesicles derived from grapefruit, ginger, or purple cabbage have been utilized to transport Dox and nucleic acid microRNA (miRNA) for tumor treatment (Zhuang et al., 2016; Zhang et al., 2016; You et al., 2021).

The vesicles derived from Chinese medicine have garnered significant research interest due to their pharmacological effects. Peng et al. were the first to isolate exosome-like nanovesicles from Brucea javanica, a traditional Chinese medicine. Their study found that these nanovesicles were capable of efficiently delivering 10 functional miRNAs to tumor cells, thereby inducing tumor cell apoptosis through the PI3K/Akt/mTOR signaling pathway and ROS/Caspase mechanisms. This process ultimately inhibited the growth, metastasis and angiogenesis of breast cancer (Yan et al., 2024). Cao’s research involved the intraperitoneal injection of nanoparticles derived from Ginseng into animal models, aiming to regulate myeloid differentiation factor 88 (MyD88) and Toll-like receptors-4 (TLR4) mediated signal transduction, and significantly inhibit melanoma growth (Cao et al., 2019). Subsequent research has demonstrated that Ginseng-derived exosome-like nanoparticles potentiate the anti-tumor efficacy of PD-1 monoclonal antibodies by activating tumor-infiltrating T lymphocytes. This mechanism presents a straightforward approach to modulating the cold tumor microenvironment and augmenting PD-1 monoclonal antibody-based immunotherapy (Han et al., 2022). The consensus statement on research and application of Chinese herbal medicine-derived extracellular vesicles (2023 edition) has articulated 13 consensus opinions, offering a valuable reference for the development of Chinese herbal vesicles. This statement underscores the extensive research efforts and significant potential of these vesicles in disease treatment (Zhao et al., 2023). In conclusion, PNVs, characterized by their strong expanded production capacity and minimal immunogenicity, represent a promising platform for tumor therapy. However, for clinical application, precise extraction and purification methodologies must be established. Additionally, the mechanism and clinical efficacy of PNVs warrant thorough investigation. Further clinical trials are imperative to validate their therapeutic potential.

### 2.4 EVs from animal

EVs derived from animals are predominantly focus on milk-derived exosomes (MEs). Bovine milk, which is abundant in EVs, readily accessible, and safe for use, represents a significant source of EVs (Ngu et al., 2022). In a study conducted by Chen Ying et al., MEs were employed as delivery vehicles and were further modified with the M2 macrophage-binding peptide M2pep and the anti-EGFR antibody 7D12. This engineered system demonstrated the capability to specifically deliver siPD-L1 to M2 tumor-associated macrophage (TAM), thereby inhibiting PD-L1 expression, restoring CD8+T cell activity, reconfiguring the immunosuppressive tumor microenvironment, and exerting anticancer effects (Chen et al., 2024). MEs encapsulating anthocyanidins were administered orally and showed the ability to target colon cancer, resulting in a reduction in the number of colon tumors in a mouse model (Mudd et al., 2020). In comparison to control groups, paclitaxel-loaded bovine MEs reduced cancer growth by 60% when administered orally to a mouse model. This delivery system addressed the limitations of paclitaxel, including poor solubility, and mitigated hepatic, renal and systemic toxicity (Agrawal et al., 2017). Additionally, MEs were employed to deliver exogenous hsa-miR148a-3p to HepG2 and Caco-2 cancer cells, exerting a biological effect by modulating gene expression (Del Pozo-Acebo et al., 2021). The pharmaceutical industry has acknowledged and licensed the use of MEs for the delivery of RNA therapeutics to brain tumors (Ngu et al., 2022). MEs represent a cost-effective vesicle-based drug delivery platform and serve as a viable alternative to oral drug administration. However, it is important to note that MEs may pose potential health risks due to the presence of miRNAs, which have been associated with conditions such as type 2 diabetes and obesity. Additionally, certain proteins within MEs may act as allergens, thereby raising biosafety concerns (Zhong et al., 2023). Consequently, rigorous purification protocols are essential for the safe application of MEs.

### 2.5 EVs from bacteria

Bacterial extracellular vesicles (BEVs), derived from Gram-positive bacteria or Gram-negative bacteria, have emerged as an emerging platform for tumor treatment. These vesicles encompass a variety of components, such as proteins, lipids, lipoproteins, DNA, and RNA (Xie et al., 2023). Outer membrane vesicles (OMVs) derived from photosynthetic bacteria are enriched with bioactive constituents of the parent cells. OMVs have the ability to polarize macrophages, activate dendritic cells, enhance antigen presentation, and exhibit significant antitumor activity in tumor models (Xiao et al., 2024). Engineered BEVs can be designed for the development of antibacterial and anticancer vaccines. For instance, a manganese peroxide interface was applied to encapsulate vesicles derived from tumor-symbiotic bacteria. Upon uptake by DC cells, the intracellular lysosome degraded the manganese oxide shell. The released bacterial vesicles subsequently activated humoral immunity by promoting the release of immunoglobulin G (IgG) from B cells, thereby effectively inhibiting the proliferation of tumor-symbiotic bacteria. Simultaneously, manganese ions activated the cGAS-STING pathway, facilitating the maturation of DC cells. This process enabled DC cells to present specific antigens to T cells, thereby enhancing the immune response (Zhang et al., 2024). Genetically engineered bacterial protoplast nanovesicles, produced through extrusion, had potential applications in gene delivery for tumor treatment. These vesicles encapsulated gene-edited ribonucleoprotein (RNP) capable of targeting Pik3cg, thereby promoting the polarization of macrophages towards the M1 phenotype. The bacterial DNA within the vesicles could activate macrophage Toll-like receptor 9 (TLR9), sustaining the proinflammatory state of the macrophages. Additionally, vesicles modified with polyethylene glycol (PEG)-galactosamine could specifically target tumor-associated macrophages, facilitating anti-tumor immunization (Zhao et al., 2024). Rich in bacterial antigens, these vesicles also serve as potent vaccine adjutants, enhancing the immune response. For instance, bacteria-derived vesicles containing the STING agonist cyclic GMP-AMP (cGAMP) were conjugated to denucleated melanoma cells via a click chemistry reaction to generate a live tumor cell vaccine. This vaccine effectively activated the STING pathway, promoted the maturation and activation of DC cells, and polarized M1 macrophages, thereby significantly inhibiting melanoma progression. Furthermore, this vaccine had the potential to enhance the tumor immune microenvironment by facilitating the infiltration of immune cells and promoting the proliferation and activation of tumor-specific T cells, thereby substantially improving the therapeutic efficacy of PD-1 antibody treatment (Fang et al., 2024).

The aforementioned findings suggest that BEVs possess potential therapeutic applications in cancer treatment. However, it is important to note that not all BEVs are suitable candidates for tumor therapy. For instance, EVs derived from methicillin-resistant *Staphylococcus aureus* (MRSA) have been shown to stimulate the production of Th-2-ralated cytokines, resulting in anaphylactic shock in murine models. This indicates that such BEVs can elicit IgE-mediated systemic hypersensitivity reactions (Asano et al., 2021). OEVs secreted by *Pseudomonas aeruginosa* and *Escherichia coli* have been shown to non-specifically reduce mitochondrial membrane potential, thereby inducing apoptosis and inflammation through the activation of B cell lymphoma 2 (BCL-2)-associated x protein (BAX) and BCL-2 antagonist killer (BAK) proteins and the downregulation of the pro-survival protein BCL-2 (Deo et al., 2020). The biosecurity implications of BEVs remain inadequately understood and warrant further investigation.

## 3 Production of extracellular vesicles

EVs can be isolated from cell culture supernatants or various biological fluids. However, the endogenous production of EVs presents several challenges, such as the limited yield of vesicles secreted by cells within a short time, the absence of standard preparation protocols, and the potential contamination with proteins. Additionally, EVs isolated using different methodologies exhibit heterogeneous characteristics and functional properties, complicating their large-scale production and clinical application. Moreover, the targeted efficiency of EVs towards tumor cells remains suboptimal (Du et al., 2022). Due to the precision and controllability inherent in the production process and the final product, it is anticipated that EV analogs will address these challenges. However, it is crucial to consider the toxicity associated with the residues of synthetic materials during the preparation process (Liang et al., 2021). The subsequent sections will discuss the production of EVs, EV analogs, and their further modifications in detail.

### 3.1 EVs production

**FIGURE 2:**
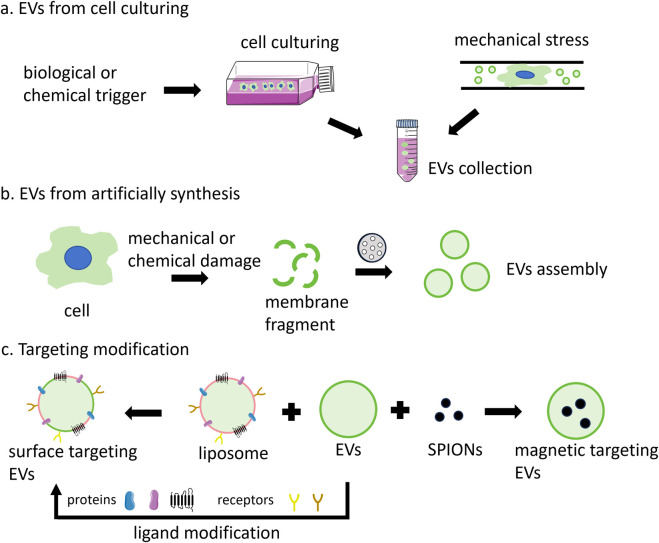
The main production methods of EVs include: **(A)** secretion by cells under natural conditions or through external induction; **(B)** assembled by the cell membrane; and **(C)** targeted vesicle production through the fusion of EVs with functional liposomes, surface modification with various ligands, or magnetic guidance using SPIONs.

The predominant approach involves the collection of EVs secreted by cells in the culture medium (Figure 2A). Initially, serum is subjected to ultracentrifugation at 100,000 g for several hours to eliminate existing vesicles. Subsequently, cells are cultured in a complete medium supplemented with vesicle-depleted serum. The culture medium is then harvested for the subsequent isolation of released EVs. However, EVs obtained through this method exhibit low yield and purity, along with a biased size distribution (Piffoux et al., 2017; Wang et al., 2022). The release of EVs can be augmented by external stimuli, including biological (starvation, hypoxia, or cytokine activation), chemical (cytochalasin B), and physical triggers (mechanical stress) (Piffoux et al., 2019). For instance, starvation has been shown to induce cell stress and subsequent EVs release. So, EVs can be produced from cells after culturing in serum-free basic medium. This production method is relatively efficient, yielding EVs of small size, high purity, and substantial quantity (Stolk and Seifert, 2015; Debbi et al., 2022). Additionally, a microfluidic device has been developed to apply mechanical stress to cells, thereby enhancing EV release. In this process, cells were suspended in a buffer and extruded at 4°C through fabricated microchannels using a syringe. EVs could then be harvested from the extrusion medium. While this method facilitates rapid EV production within hours, it is also destructive to cells, resulting in a high number of debris and apoptotic bodies (Piffoux et al., 2017). Rui Hao et al. have developed a microfluidic device featuring a series of narrow squeezing ridges. Mesenchymal stem cells traversed the channel rapidly and secreted small EVs in response to mechanical stimulation. This device has the potential to enhance EV secretion by approximately 4-fold (Hao et al., 2023).

### 3.2 EV analogs production

The lack of standardized methods for EVs production represents a significant barrier to their broader application. The inherent heterogeneity and complex composition of EVs further contribute to potential safety concerns. EVs produced through artificial synthesis offer high yield and uniform size, devoid of unwanted components, thereby garnering increasing attention (Liu Y. et al., 2019). EVs analogs, constructed using cell-derived plasma membrane fragments, can substantially enhance yield and reduce production time while maintaining a relatively uniform size (Figure 2B). The protein composition of these EV analogs more closely resembles that of the source cell compared to natural EVs. A top-down strategy has been employed to generate EV analogs (Raza et al., 2021). This process involves the initial removal of unwanted components from the cell culture medium or plasma via centrifugation, followed by the mechanical or chemical destruction of cells to isolate the cell membrane and other organelles. Finally, the cell membrane is obtained through differential or gradient centrifugation. The size of the cell membrane is ascertained using porous polycarbonate membranes. Typically, cells are sequentially passed through membrane filters with varying pore sizes. Besides, the cell membrane can be extracted through a series of steps, including hypoosmotic treatment, homogenization, ultrasonic treatment, freeze-thaw cycles, filtration, and polyethylene glycol treatment. Subsequently, EV analogs are prepared, resulting in a 50–100-fold increase in yield and a significant reduction in production cost (Rayamajhi et al., 2019).

### 3.3 Modification of EVs

The limited targeting ability of EVs, particularly exosomes, constrains their application. Further modifications can enhance the efficacy of these vesicles (Figure 2C). By fusing EVs with synthetic liposomes, hybrid EV analogs can be generated to accommodate a broader range of applications (Du et al., 2022). For example, isolated small cell vesicles were added into the aqueous phase to hydrate the phospholipid membrane. Subsequently, the fused exosomes were prepared through extrusion, which combined the multiple advantages of endogenous cell vesicles with the ease of liposome surface modification (Wen et al., 2022). EVs derived from macrophages and cRGD particles were incubated to produce cRGD-modified EVs. In this context, macrophage-derived EVs inherit the tumor-targeting capabilities of macrophages, while cRGD also contribute to tumor cell targeting, thereby achieving a dual-targeting effect (Huang et al., 2022).

Furthermore, the integration of magnetic materials with EVs can augment their targeting efficacy. Superparamagnetic iron oxide nanoparticles (SPIONs) with magnetic navigation can be loaded into EVs through various techniques, such as electroporation, natural incubation, cell extrusion, or post-modification bonding with ligands (Zhuo et al., 2021). EVs loaded with SPIONs and therapeutic agents such as Dox or tumor necrosis factor-alpha (TNF-α) have demonstrated improved targeting and tumor inhibition when subjected to applied magnetic fields (Qi et al., 2016; Zhuang et al., 2020). Additionally, SPION-loaded exosomes can serve as agents for magnetic hyperthermia therapy (MHT), facilitating tumor cell eradication, and as contrast agents for tumor magnetic resonance imaging (MRI) diagnostics (Altanerova et al., 2017).

In addition to preserving source cell proteins during preparation for targeted delivery via protein-protein interactions, surface modification of EVs with targeting moieties can enhance recognition-mediated targeting. Surface targeting modification can be realized by genetic engineering or chemical modification. For the former, plasmids were engineered to encode homing peptides or ligands fused with transmembrane proteins. Subsequently, donor cells transfected with these plasmids were capable of secreting engineered vesicles that displayed targeted groups on their surfaces (Zhu et al., 2022; Chiang et al., 2023). The fusion of the αγ integrin-specific iRGD with LAMP-2B enabled exosomes to deliver Dox to integrin-positive breast cancer cells (Jia et al., 2018). GE11(YHWYGYTPQNVI) was genetically fused to the transmembrane protein platelet-derived growth factor receptor (PDGFR), resulting in the production of GE11 exosome, which exhibited high affinity for EGFR-overexpressing cancer cells (Ohno et al., 2013). Chemical modification is another way to achieving targeted functionality in vesicles. In this context, proteins present on the vesicle surface serve as anchors for attaching chemical groups and targeted molecules. For instance, an alkyne group was conjugated to a protein on the exosome membrane via an EDC-NHS condensation reaction. Subsequently, an RGE-peptide containing an azido group was linked to the alkyne group through triazole linkages. These glioma-targeted exosomes were capable of crossing the blood-brain barrier and inhibiting glioma progression (Jia et al., 2018). In addition, surface modification can be accomplished by synthesizing amphiphilic molecules with specific targeting groups and incorporating them into vesicles. For example, aminoethyl anisamide (AA) linked DSPE-PEG was conjugated to the exosome membrane, resulting in enhanced paclitaxel (PTX) uptake in lung cancer cells (Kim et al., 2018).

## 4 Purification of extracellular vesicles

The commonly employed methods for separation and purification encompass centrifugation, filtration, chromatography, polymer coprecipitation, and microfluidic technology, among others (Piffoux et al., 2017; Zhu et al., 2020). Large vesicles can be obtained through conventional centrifugal operations. Exosomes, due to their minimal size, necessitate specialized purification techniques, such as ultracentrifugation and polymer coprecipitation. Various separation methods have been categorized based on distinct separation principles (Table 2).

**TABLE 2:** EVs separation methods.

Method	Diagram	Principle	Advantage	Disadvantage	Refs
Ultracentrifugation	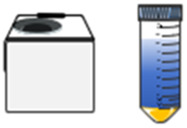	Density and size differences	Large sample volume, relatively mature, do not need additional separation reagents	Time-consuming, low purity, risk of EV damage, high cost with expensive equipment	Johnstone (1992), Konoshenko et al. (2018)
Ultrafiltration	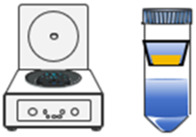	Size differences	Short time-consuming, easy to operate	Clog membrane pores, risk of EV damage, exosomes attach on membrane	Xiang et al. (2021), McNamara et al. (2018)
Size-exclusion chromatography	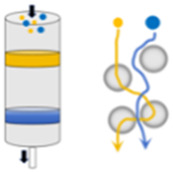	Size differences	High purity, unbroken exosome activity, moderate sample volume	Take a long time, require special equipment	Xu et al. (2016), Guo et al. (2021)
Immunoaffinity chromatography	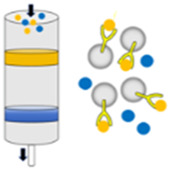	Membrane surface antigens bind specifically to receptors	High purity, suitable for specific exosomes	High cost, low yield, need to establish label antibodies, only for cell-free samples	Park et al. (2021), Zhang et al. (2018)
Polymer coprecipitation	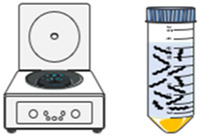	Polymer changes solubility to precipitate	Easy to operate, large sample volume	Take a long time, co-precipitation with other non-exosomal substances	Yang et al. (2020), Martínez-Greene et al. (2021)
Microfluidics	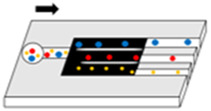	Size, fluid dynamics, immunoaffinity	Easy to operate, fast, intelligent	Lack of large-scale testing and standardization, low sample volume	Tong et al. (2024), Bai et al. (2023)

### 4.1 Separation according to density

Differential centrifugation is performed under varying centrifugal forces, leveraging the differential densities of EVs in the sample. The samples are derived from a diverse array of sources, including cell supernatant, serum, saliva, urine, and cerebrospinal fluid, among others (Yang et al., 2020). The liquid samples undergo a series of centrifugation steps: initially at 300 g for 10 min to eliminate dead cells, subsequently at 2,000 g for 10 min to remove cell debris, followed by centrifugation at 10,000 g for 30 min to exclude large vesicles, and finally at 120,000 g for 90 min to isolate small EVs (Zhu et al., 2022). Johnstone pioneered the use of ultracentrifugation (UC) for the extraction of exosomes from reticulocyte culture medium (Johnstone, 1992). Currently, this technique is wildly regarded as the “gold standard” for exosome isolation. The dead cells, cell fragments, and large vesicles were initially re-moved via low-speed centrifugation. Subsequently, exosomes were isolated through UC at 100,000 g. This method is well-established and does not require the addition of supplementary separation reagents. However, it has several limitations, including low specificity, poor reproducibility, the aggregation and fragmentation of exosomes, increased contamination with soluble protein impurities, and high costs associated with the use of expensive equipment (Gardiner et al., 2016; Konoshenko et al., 2018).

Density gradient centrifugation isolates exosomes based on differences in particle size and density. Typically, sucrose is employed as the medium to establish a density gradient layer that progressively increases from the upper to the lower strata. When subjected to centrifugal force, exosomes are ultimately distributed within a density range of 1.10–1.18 g/mL. The exosomes obtained through this method are largely devoid of extraneous proteins. However, the equipment required for this process is costly, and prolonged centrifugation can compromise the structural integrity of the exosomes, rendering it un-suitable for processing large sample volumes (Zhu et al., 2020; Onódi et al., 2018).

### 4.2 Separation according to sizes

Filtration separation techniques are predicated on particle size differentiation. For example, ultrafiltration is achieved by obstructing particles larger than 200 nm in diameter from passing through the membrane pores, while allowing smaller vesicles, typically less than 20 nm in diameter, to permeate. This process can be expedited through pressurization or brief periods of low-speed centrifugation. The selection of vesicles of specific sizes is facilitated by the diameter of the membrane pores, often serving as an auxiliary step in ultrafast centrifugation methods. However, the membrane pores are prone to blockage, and exosomes are susceptible to damage during the process (Xiang et al., 2021). The tangential flow filtration (TFF) method alters the flow direction of the liquid from perpendicular to parallel relative to the filter membrane. This adjustment mitigates exosome damage, reduces membrane pore clogging, and extends the service life of the filter membrane. Consequently, TFF is suitable for the industrial preparation of vesicles (McNamara et al., 2018).

Size-exclusion chromatography (SEC) is employed based on differential particle sizes. During SEC, vesicles traverse the separation matrix and are eluted using phosphate-buffered saline (PBS). Larger particles or molecules, unable to penetrate the separation matrix, are rapidly and directly eluted. Small volume particles may infiltrate the matrix, resulting in prolonged retention times and delayed elution. EVs are collected at various elution times. Commonly utilized substrates include Sephadex, Sepharose, and Sephacryl. The process is gravity-driven, ensuring the integrity of the exosomes and yielding a high-purity product however, the eluent exhibits a dilutive effect (Xu et al., 2016; Guo et al., 2021).

### 4.3 Separation based on immunoaffinity

Immunoaffinity chromatography leverages specific antigens present on the vesicle surface. Exosomes are isolated through solid-phase antibody adsorption, a method that holds potential for disease diagnosis. Typically, exosome surface biomarkers, such as the four-transmembrane protein superfamily, are employed as ligands in this process. Anti-bodies can be adsorbed onto magnetic beads, porous silica monoliths, or membrane affinity filters. Exosomes can be isolated through the specific binding of their surface proteins to a solid phase during the passage of the solution. For instance, exosomes can be extract-ed by targeting the exosome surface protein CD81 with antibodies or by utilizing the interaction between the Tim4 protein immobilized on magnetic beads and phosphatidyl-serine (PS) on the exosome surface. This method achieves high extraction efficiency within a short duration. However, the potential impact on the biological function of the extracted exosomes warrants further investigation (Park et al., 2021; Zhang et al., 2018).

### 4.4 Polymer coprecipitation method

The polymer coprecipitation method employs PEG as the separation medium. In this separation process, PEG interacts with the water molecules on the exosome surface, thereby reducing their solubility and facilitating sedimentation separation. Initially, the sample to be separated is incubated with PEG (the molecular weight is 8,000Da) at 4°C for a duration of 8–16 h. Subsequently, exosomes are isolated through low-speed centrifugation. At present, the associated kits are user-friendly and exhibit a high acquisition rate, making them suitable for large-scale exosome extraction. However, it is important to note that PEG not only precipitates exosomes but also co-precipitates hydrophilic nucleic acids, lipoproteins, and other components, resulting in lower purity (Yang et al., 2020; Martínez-Greene et al., 2021).

### 4.5 Separation by microfluidics

Microfluidics offers significant potential for the separation and real-time detection of EVs due to its inherent advantages, including low sample consumption, rapid reaction times, and precise fluid control. The primary methods employed in the context are based on immune affinity capture and size separation, and also encompass techniques such as membrane filtration, on-chip microstructures, the application of acoustic or electric fields, and the utilization of viscoelastic flow characteristics (Salaf et al., 2016; Yang et al., 2017). Despite these advancements, there remains considerable scope for enhancing the degree of automation and reducing processing time. Hongju Mao et al. Have introduced an EVs processing platform based on digital microfluidic technology, achieving a capture efficiency of 77% within 20 min (Tong et al., 2024). The ExoTIC device demonstrates superior efficacy in isolating exosomes from bodily samples compared to PEG precipitation and UC (Lin et al., 2020). Utilizing a viscoelastic-based microfluidic platform, small cell vesicles could be isolated from whole blood without the need for labels, achieving purity and recovery rates of 97% and 87%, respectively (Meng Y. et al., 2023). Additionally, a dean-flow-coupled elasto-inertial microfluidic chip has been developed to purify exosomes from cell culture medium and human serum, yielding a 70.6% recovery rate and a 91.4% protein removal rate (Bai et al., 2023).

## 5 Drug loading for cancer therapy

**FIGURE 3:**
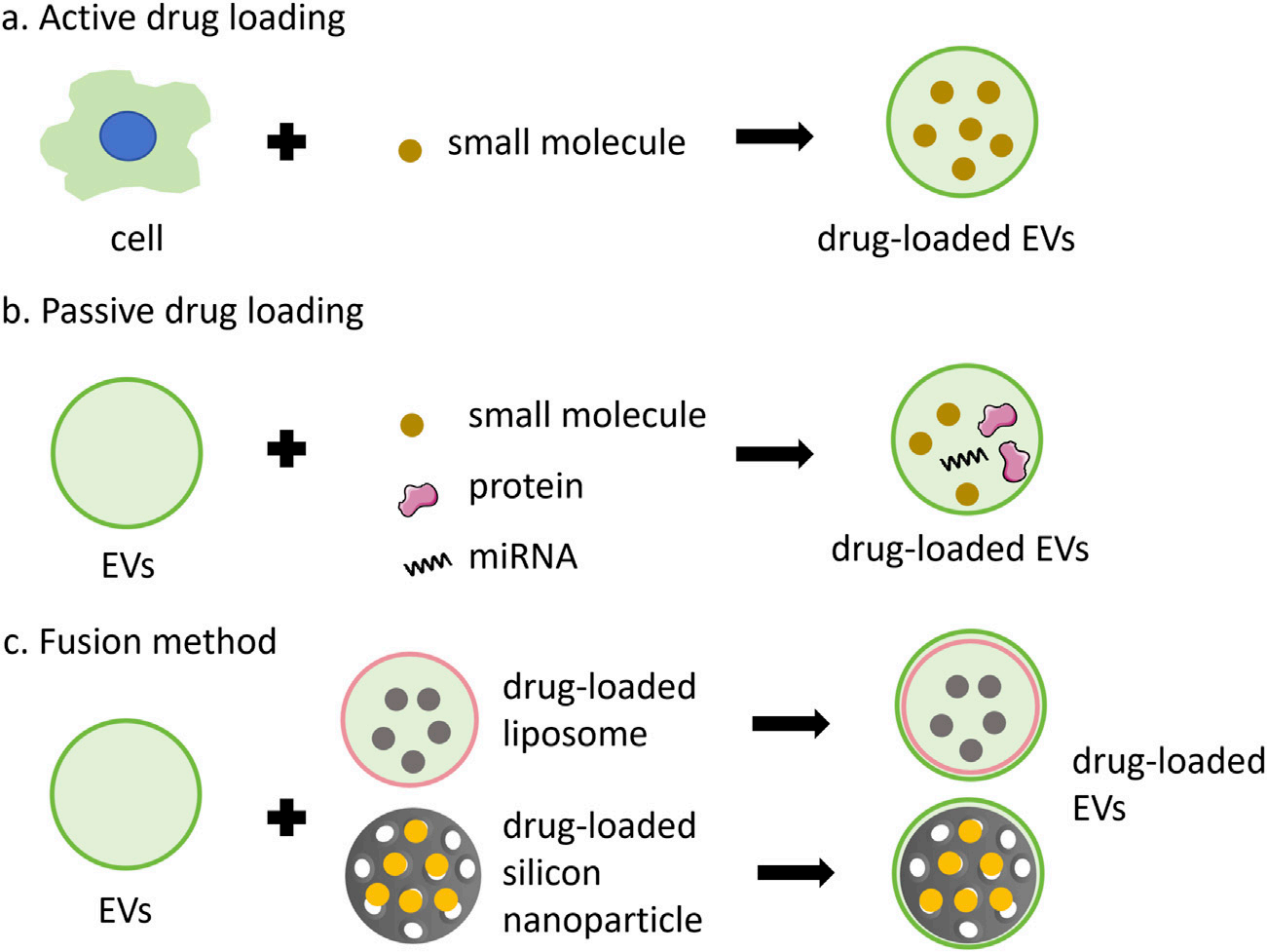
Anti-cancer drugs can be encapsulated into EVs through various methods. **(A)** active loading leverages the cell’s biological activity to carry small molecules, such as PTX, Dox and 5FU; **(B)** passive loading involves techniques such as incubation, extrusion, ultrasound, or electroporation to incorporate small molecules, proteins, or miRNA; **(C)** the fusion method combines the advantages of nanoparticles and EVs.

The therapeutic application of EVs in drug delivery systems encompasses both direct and indirect effects in tumor treatment. Given that EVs are derived from their parent cells, they retain the functional properties of these cells and can exert direct anti-tumor effects. Besides, the release of encapsulated drugs, which can play a direct anti-tumor effect. Indirect effects involve the modulation of tumor gene expression or regulation of the systemic immune response by EVs. Strategies for incorporating anti-cancer drugs into EVs can be classified into active or passive methods (Figures 3A,B).

The active method involves extracting EVs from the medium following the incubation of drugs with cells. For example, EVs derived from the conditioned medium of SR4987 cells incubated with PTX have shown promising efficacy against tumor cells (Pascucci et al., 2014). However, this method is generally inefficient and time-consuming. To enhance the method, cells incubated with drugs can be mechanically squeezed to obtain drug-carrying EVs. Such as, exosome-like vesicles loaded with drugs were produced by continuously squeezing mononuclear cells/macrophages together with Dox, 5-fluorouracil (5FU), gecitabine, and carboplatin, which demonstrated the ability to reduce tumor growth without significant adverse reactions *in vivo* (Jang et al., 2013). The active method is suitable for loading small molecules or proteins.

Passive drug packaging into EVs is achieved through incubation, extrusion, ultrasound, or electroporation after vesicle acquisition (Xie et al., 2020). This approach can be employed to deliver chemotherapy drugs, nucleic acids, proteins, and other anti-tumor agents. For instance, after incubating syncretic vesicles with Dox, drug-carrying vesicles were prepared sequentially by vortex mixing, ultrasound treatment, and extrusion through 200 nm polycarbonate membranes. This process enhances the tumor cytotoxicity of Dox (Wen et al., 2022). Besides, vesicles containing two drugs were prepared through 200 nm polycarbonate membranes, along with macrophage cell membrane, methyltransferase-like 14 (METTL14), and the toll-like receptor 4 agonist RS09. These vesicles were employed to inhibit osteosarcoma and macrophage polarization (Huang et al., 2022). Most miRNAs can be encapsulated into EVs via electroporation. For instance, EVs and miRNAs could be mixed and subjected to electroporation with a 20 ms pulse at varying voltages and pulse numbers. The mixture was then incubated for a specified period, and the miRNA-loaded EVs were subsequently obtained by ultracentrifugation. However, this method may compromise the structural integrity of the drugs and the vesicles (Pomatto et al., 2019; Taghikhani et al., 2019). Small molecule drugs, such as Dox or curcumin, can also be encapsulated within EVs via electroporation (Zhu et al., 2022; Li et al., 2020; Wang et al., 2020).

Additionally, drug loading can be accomplished vis fusion method (Figure 3C). Fusion EVs integrate the benefits of synthetic nanoparticles and multiple EVs, offering a safer and more efficacious drug delivery system. The encapsulation of synthetic nanoparticles within EV membranes can shield the drugs from immune clearance and facilitate intra-cellular drug release (Liu C. et al., 2019; Cheng et al., 2018). Zhang et al. (2019) Engineered a bionic chimeric vesicle-based drug delivery system by integrating cell membrane proteins from red blood cells and breast cancer cells with Dox-containing liposomes. This system demonstrated the potential to enhance drug concentration at tumor sites, reduce hepatic retention, and improve therapeutic efficacy against cancer. Similarly, Yong et al. utilized tumor cell-EVs to coat Dox-containing porous silicon nanoparticles, resulting in drug carriers with high tumor accumulation and permeability. This approach exhibited enhanced anti-tumor activity and efficacy in eradicating cancer stem cells across various cancer models (Yong et al., 2019). The results indicate that utilizing engineered EVs for the delivery of anti-cancer drugs offers numerous advantages, including enhanced homologous targeting capabilities, diminished uptake by mononuclear and macrophage cells, and favorable therapeutic outcomes in primary, metastatic, and drug-resistant cancers. Fusion methods exhibit attributes such as mass production, convenient preparation, and a controllable manufacturing process, showing the significant potential of fusion vesicles for clinical applications as substitutes for natural EVs (Zhang X. et al., 2021).

## 6 Future prospects and conclusion

Endogenous EVs possess the capability to evade recognition by the mononuclear phagocyte system, thereby reducing the immunogenicity of therapeutic agents, overcoming biological barriers, enhancing drug accumulation at tumor sites, minimizing adverse effects, activating anti-tumor immune responses, and ultimately improving anti-tumor efficacy. Consequently, EV-based drug delivery systems represent promising strategies for tumor treatment (Table 3). Therein, the primary advantage of PNVs as an emerging modality for cancer treatment lies in their inherent non-toxicity. Examples include plant exosomes loaded with curcumin (NCT01294072) and grape exosomes used in conjunction with chemoradiation (NCT01668849). Additionally, PNVs exhibit greater production capacity compared to other EVs, rendering them a safer and more cost-effective therapeutic option. Nonetheless, the clinical translation and application of EV-based cancer therapies remain in nascent stages.

**TABLE 3:** Ongoing clinical trials of EV-based cancer therapeutics at www.clinicaltrials.gov.

Name	Status	EVs	Cancer	Location	NCT number
A study of exoASO-STAT6 (CDK-004) in patients with advanced hepatocellular carcinoma (HCC) and patients with liver metastases from either primary gastric cancer or colorectal cancer (CRC)	Phase I	exoASO-STAT6: cell-derived exosomes loaded with a synthetic lipid-tagged oligonucleotide	Advanced hepatocellular carcinoma, primary gastric cancer or colorectal cancer with secondary liver metastases	United States	05375604
Study investigating the ability of plant exosomes to deliver curcumin to normal and colon cancer tissue	Phase I	Curcumin conjugated with plant exosomes	Colon Cancer	United States	01294072
iExosomes in treating participants with metastatic pancreas cancer with KrasG12D mutation	Phase I	Mesenchymal Stromal Cells-Derived Exosomes with KrasG12D siRNA	Metastatic Pancreas Cancer Patients Harboring KrasG12D Mutation	United States	03608631
An open, dose-escalation clinical study of chimeric exosomal tumor calcines for recurrent or metastatic bladder cancer	Phase I	Chimeric exosomal tumor vaccines	Recurrent or Metastatic Bladder Cancer	China	05559177
Edible plant exosome ability to prevent oral mucositis associated with chemoradiation treatment of head and neck cancer	Phase I	Grape exosomes	Head and Neck Cancer	United States	01668849

Current EV extraction technologies predominantly rely on principles of volume and density differentiation, which are primarily utilized in pre-clinical basic research. The transition from scientific experimentation to the commercialization of EVs necessitates careful consideration of cell culture, EV extraction, and purification processes. Bioreactor technology and perfusion culture methods are pivotal for achieving large-scale production. Investigating the impact of optimal culture conditions, including growth factors, oxygen concentration, and other stimuli, is essential for enhancing production efficiency. Furthermore, the clinical application of EVs demands rigorous assessment of their reproducibility, scalability, stability, and performance in allogeneic environments. It is also important to note that different extraction methods may yield varying therapeutic effects. Therefore, establishing a standardized protocol for the extraction and purification of EVs specific to a given disease is of paramount importance. Minimum Information for Studies of Extracellular Vesicles (MISEV) 2023 guidelines offer comprehensive information pertinent to EV research (Welsh et al., 2024). Scholars have deliberated on the latest advancements in rapid EV separation technologies and the integration of multiple separation methodologies. When extracting EVs from diverse sample types, several critical factors must be considered, including the specific source of the sample, the subpopulation of EVs, the desired yield, and the downstream analytical methods to be employed. Currently, no single method exists that is universally applicable for the analysis of all sample types and purposes. To enhance the quantification of EV purity, the accuracy of purity characterization can be improved through the utilization of orthogonal methods and the application of specific ratios, such as the protein-to-lipid ratio, protein-to-particle ratio, or RNA-to-particle ratio. Despite advancements toward the standardization of extraction technologies, challenges remain in establishing comprehensive standards for EV collection and separation, unifying identification criteria, and standardizing the product preparation processes. These issues necessitate further clarification and resolution in future research.

Another significant concern regarding EVs is their targeting efficacy towards tumor cells. Wilhelm and colleagues conducted an extensive meta-analysis of various nanomaterials (NMs), revealing that the targeting efficiency of NMs to solid tumors is markedly limited, with an average of only 0.7% of the injected dose reaching the tumor site (Wilhelm et al., 2016). For antibody-modified NMs, the targeting efficacy was notably low, with only 2% of cancer cells being affected by the drugs. This limited efficacy is attributed to the entrapment of NMs in the extracellular matrix or their sequestration by tumor-associated macrophages (Dai et al., 2018). How about the targeting efficiency of EVs and EV analogs to tumors *in vivo*? It is still an area requiring extensive and thorough investigation. Effective surface modification is essential for achieving precision and controllability in the targeted therapy of EVs. Additionally, a comprehensive consideration of factors such as circulation time, tumor penetration, cellular internalization, and drug release is necessary, as these factors are interdependent and may influence one another. The progress and application of synthetic biotechnology enable the customized design and synthesis of engineered vesicles, thereby allowing precise control over their properties and enhancing their therapeutic performance.

The Food and Drug Administration (FDA) has reported serious adverse effects experienced by patients following the use of drugs containing exosomes, indicating significant safety concerns for the application of EV-based therapeutics (Herrmann et al., 2021). Consequently, several production challenges of EVs must be addressed, including the establishment of a stable source, the development of standardized production processes, and the implementation of reliable quality control measures. Furthermore, additional preclinical and clinical studies are necessary to bridge the gap between the ideal and the actual application of EVs. These studies cover factors such as the administration routes and dosages of EVs for various tumors and patient populations, long-term safety, and the risk-benefit ratio in comparison to liposome-based alternatives. The entire process of EV production, characterization, efficacy, and safety must be reported transparently to foster a positive developmental environment. Despite numerous challenges, the significant advantages of biogenic vesicles, coupled with the collaborative efforts of researchers worldwide, position EV-based drug delivery platforms to realize substantial potential in cancer treatment.

## References

[B1] Aarsund M. Segers F. M. Wu Y. Inngjerdingen M. (2022). Comparison of characteristics and tumor targeting properties of extracellular vesicles derived from primary NK cells or NK-cell lines stimulated with IL-15 or IL-12/15/18. Cancer Immunol. Immunother. 71, 2227–2238. 10.1007/s00262-022-03161-0 35119498 PMC9374793

[B2] Agrawal A. K. Aqil F. Jeyabalan J. Spencer W. A. Beck J. Gachuki B. W. (2017). Milk-derived exosomes for oral delivery of paclitaxel. Nanomedicine 13, 1627–1636. 10.1016/j.nano.2017.03.001 28300659

[B3] Altanerova U. Babincova M. Babinec P. Benejova K. Jakubechova J. Altanerova V. (2017). Human mesenchymal stem cell-derived iron oxide exosomes allow targeted ablation of tumor cells via magnetic hyperthermia. Int. J. Nanomedicine 27 (12), 7923–7936. 10.2147/IJN.S145096 PMC5667789 29138559

[B4] Asano K. Hirose S. Narita K. Subsomwong P. Kawai N. Sukchawalit R. (2021). Extracellular vesicles from methicillin resistant Staphylococcus aureus stimulate proinflammatory cytokine production and trigger IgE-mediated hypersensitivity. Emerg. Microbes Infect. 10, 2000–2009. 10.1080/22221751.2021.1991239 34623928 PMC8547819

[B5] Attarilar S. Yang J. Ebrahimi M. Wang Q. Liu J. Tang Y. (2020). The toxicity phenomenon and the related occurrence in metal and metal oxide nanoparticles: a brief review from the biomedical perspective. Front. Bioeng. Biotechnol. 8, 822. 10.3389/fbioe.2020.00822 32766232 PMC7380248

[B6] Bai J. J. Zhang X. Wei X. Wang Y. Du C. Wang Z. J. (2023). Dean-flow-coupled elasto-inertial focusing accelerates exosome purification to facilitate single vesicle profiling. Anal. Chem. 95, 2523–2531. 10.1021/acs.analchem.2c04898 36657481

[B7] Bao S. Zheng H. Ye J. Huang H. Zhou B. Yao Q. (2021). Dual targeting EGFR and STAT3 with Erlotinib and Alantolactone co-loaded PLGA nanoparticles for pancreatic cancer treatment. Front. Pharmacol. 12, 625084. 10.3389/fphar.2021.625084 33815107 PMC8017486

[B8] Bobo D. Robinson K. J. Islam J. Thurecht K. J. Corrie S. R. (2016). Nanoparticle based medicines: a review of FDA-approved materials and clinical trials to date. Pharm. Res. 33, 2373–2387. 10.1007/s11095-016-1958-5 27299311

[B9] Bose R. J. C. Uday Kumar S. Zeng Y. Afjei R. Robinson E. Lau K. (2018). Tumor cell-derived extracellular vesicle-coated nanocarriers: an efficient theranostic platform for the cancer-specific delivery of anti-miR-21 and imaging agents. ACS Nano 12, 10817–10832. 10.1021/acsnano.8b02587 30346694 PMC6684278

[B10] Bray F. Laversanne M. Sung H. Ferlay J. Siegel R. L. Soerjomataram I. (2024). Global cancer statistics 2022: GLOBOCAN estimates of incidence and mortality worldwide for 36 cancers in 185 countries. CA Cancer J. Clin. 74, 229–263. 10.3322/caac.21834 38572751

[B11] Burnett M. Abuetabh Y. Wronski A. Shen F. Persad S. Leng R. (2020). Graphene oxide nanoparticles induce apoptosis in wild-type and CRISPR/Cas9-IGF/IGFBP3 knocked-out osteosarcoma cells. J. Cancer 11, 5007–5023. 10.7150/jca.46464 32742448 PMC7378933

[B12] Cao H. Gao H. Wang L. Cheng Y. Wu X. Shen X. (2022). Biosynthetic dendritic cell-exocytosed aggregation-induced emission nanoparticles for synergistic photodynamic immunotherapy. ACS Nano 16, 13992–14006. 10.1021/acsnano.2c03597 35960889

[B13] Cao M. Diao N. Cai X. Chen X. Xiao Y. Guo C. (2023). Plant exosome nanovesicles (PENs): green delivery platforms. Mater Horiz. 10, 3879–3894. 10.1039/d3mh01030a 37671650

[B14] Cao M. Yan H. Han X. Weng L. Wei Q. Sun X. (2019). Ginseng-derived nanoparticles alter macrophage polarization to inhibit melanoma growth. J. Immunother. Cancer 7, 326. 10.1186/s40425-019-0817-4 31775862 PMC6882204

[B15] Cao W. Chen H. D. Yu Y. W. Li N. Chen W. Q. (2021). Changing profiles of cancer burden worldwide and in China: a secondary analysis of the global cancer statistics. 2020. Chi. Med. J. 134, 783–791. 10.1097/CM9.0000000000001474 PMC8104205 33734139

[B16] Chen Q. Zu M. Gong H. Ma Y. Sun J. Ran S. (2023). Tea leaf-derived exosome-like nanotherapeutics retard breast tumor growth by pro-apoptosis and microbiota modulation. J. Nanobiotechnology 21, 6. 10.1186/s12951-022-01755-5 36600299 PMC9811040

[B17] Chen Y. Gong L. Cao Y. Liu Z. Wang Y. Cheng H. (2024). Reprogramming tumor-associated macrophages by a dually targeted milk exosome system as a potent monotherapy for cancer. J. Control. Release 366, 395–409. 10.1016/j.jconrel.2023.12.058 38184235

[B18] Cheng G. Li W. Ha L. Han X. Hao S. Wan Y. (2018). Self-assembly of extracellular vesicle-like metal-organic framework nanoparticles for protection and intracellular delivery of biofunctional proteins. J. Am. Chem. Soc. 140, 7282–7291. 10.1021/jacs.8b03584 29809001

[B19] Cheng Z. Li M. Dey R. Chen Y. (2021). Nanomaterials for cancer therapy: current progress and perspectives. J. Hematol. Oncol. 14, 85–27. 10.1186/s13045-021-01096-0 34059100 PMC8165984

[B20] Chiang C. L. Ma Y. Hou Y. C. Pan J. Chen S. Y. Chien M. H. (2023). Dual targeted extracellular vesicles regulate oncogenic genes in advanced pancreatic cancer. Nat. Commun. 14, 6692. 10.1038/s41467-023-42402-3 37872156 PMC10593751

[B21] Choo Y. W. Kang M. Kim H. Y. Han J. Kang S. Lee J. R. (2018). M1 macrophage-derived nanovesicles potentiate the anticancer efficacy of immune checkpoint inhibitors. ACS Nano 12, 8977–8993. 10.1021/acsnano.8b02446 30133260

[B22] Dai Q. Wilhelm S. Ding D. Syed A. M. Sindhwani S. Zhang Y. (2018). Quantifying the ligand-coated nanoparticle delivery to cancer cells in solid tumors. ACS Nano 12, 8423–8435. 10.1021/acsnano.8b03900 30016073

[B23] Debbi L. Guo S. Safina D. Levenberg S. (2022). Boosting extracellular vesicle secretion. Biotechnol. Adv. 59, 107983. 10.1016/j.biotechadv.2022.107983 35588952 PMC9420194

[B24] Del Pozo-Acebo L. Hazas M. L. L. Tomé-Carneiro J. Gil-Cabrerizo P. San-Cristobal R. Busto R. (2021). Bovine milk-derived exosomes as a drug delivery vehicle for miRNA-based therapy. Int. J. Mol. Sci. 22, 1105. 10.3390/ijms22031105 33499350 PMC7865385

[B25] Deo P. Chow S. H. Han M. L. Speir M. Huang C. Schittenhelm R. B. (2020). Mitochondrial dysfunction caused by outer membrane vesicles from Gram-negative bacteria activates intrinsic apoptosis and inflammation. Nat. Microbiol. 5, 1418–1427. 10.1038/s41564-020-0773-2 32807891

[B26] Doyle L. M. Wang M. Z. (2019). Overview of extracellular vesicles, their origin, composition, purpose, and methods for exosome isolation and analysis. Cells 8, 727. 10.3390/cells8070727 31311206 PMC6678302

[B27] Du Y. Wang H. Yang Y. Zhang J. Huang Y. Fan S. (2022). Extracellular vesicle mimetics: preparation from top-down approaches and biological functions. Adv. Healthc. Mat. 11, 2200142. 10.1002/adhm.202200142 35899756

[B28] Dubertret B. Skourides P. Norris D. J. Noireaux V. Brivanlou A. H. Libchaber A. (2002). *In vivo* imaging of quantum dots encapsulated in phospholipid micelles. Science 298, 1759–1762. 10.1126/science.1077194 12459582

[B29] Fang W. Jing Z. Li Y. Zhang Z. Lin Z. Yang Z. (2024). Harnessing enucleated cancer cells as Trojan horse cell vaccines. Cell. Rep. Phys. Sci. 5, 101752. 10.1016/j.xcrp.2023.101752

[B30] Galon J. Bruni D. (2020). Tumor immunology and tumor evolution: intertwined histories. Immunity 52, 55–81. 10.1016/j.immuni.2019.12.018 31940273

[B31] Gardiner C. Di Vizio D. Sahoo S. Théry C. Witwer K. W. Wauben M. (2016). Techniques used for the isolation and characterization of extracellular vesicles: results of a worldwide survey. J. Extracell. Vesicles 5, 32945. 10.3402/jev.v5.32945 27802845 PMC5090131

[B32] Ghosh B. Biswas S. (2021). Polymeric micelles in cancer therapy: state of the art. J. Control. Release 332, 127–147. 10.1016/j.jconrel.2021.02.016 33609621

[B33] Gong N. Sheppard N. C. Billingsley M. M. June C. H. Mitchell M. J. (2021). Nanomaterials for T-cell cancer immunotherapy. Nat. Nanotechnol. 16, 25–36. 10.1038/s41565-020-00822-y 33437036

[B34] Gui L. Ye Q. Yu L. Dou G. Zhou Y. Liu Y. (2024). Bone-targeting peptide and RNF146 modified apoptotic extracellular vesicles alleviate osteoporosis. Int. J. Nanomedicine 19, 471–488. 10.2147/IJN.S433511 38250192 PMC10800117

[B35] Guimarães D. Cavaco-Paulo A. Nogueira E. (2021). Design of liposomes as drug delivery system for therapeutic applications. Int. J. Pharm. 601, 120571. 10.1016/j.ijpharm.2021.120571 33812967

[B36] Guo J. Wu C. Lin X. Zhou J. Zhang J. Zheng W. (2021). Establishment of a simplified dichotomic size‐exclusion chromatography for isolating extracellular vesicles toward clinical applications. J. Extracell. Vesicles 10, e12145. 10.1002/jev2.12145 34514732 PMC8435528

[B37] Guo M. Wu F. Hu G. Chen L. Xu J. Xu P. (2019). Autologous tumor cell-derived microparticle-based targeted chemotherapy in lung cancer patients with malignant pleural effusion. Sci. Transl. Med. 11, eaat5690. 10.1126/scitranslmed.aat5690 30626714

[B38] Guo Z. Y. Tang Y. Cheng Y. C. (2024). Exosomes as targeted delivery drug system: advances in exosome loading, surface functionalization and potential for clinical application. Curr. Drug Deliv. 21, 473–487. 10.2174/1567201819666220613150814 35702803

[B39] Han R. Min Y. Li G. Chen S. Xie M. Zhao Z. (2023). Supercritical CO2-assisted fabrication of CM-PDA/SF/nHA nanofibrous scaffolds for bone regeneration and chemo-photothermal therapy against osteosarcoma. Biomater. Sci. 11, 5218–5231. 10.1039/d3bm00532a 37338001

[B40] Han S. Bao X. Zou Y. Wang L. Li Y. Yang L. (2023). d-lactate modulates M2 tumor-associated macrophages and remodels immunosuppressive tumor microenvironment for hepatocellular carcinoma. Sci. Adv. 9, eadg2697. 10.1126/sciadv.adg2697 37467325 PMC10355835

[B41] Han X. Wei Q. Lv Y. Weng L. Huang H. Wei Q. (2022). Ginseng-derived nanoparticles potentiate immune checkpoint antibody efficacy by reprogramming the cold tumor microenvironment. Mol. Ther. 30, 327–340. 10.1016/j.ymthe.2021.08.028 34450250 PMC8753455

[B42] Hao R. Hu S. Zhang H. Chen X. Yu Z. Ren J. (2023). Mechanical stimulation on a microfluidic device to highly enhance small extracellular vesicle secretion of mesenchymal stem cells. Mat. Today bio. 18, 100527. 10.1016/j.mtbio.2022.100527 PMC9816961 36619203

[B43] Herrmann I. K. Wood M. J. A. Fuhrmann G. (2021). Extracellular vesicles as a next-generation drug delivery platform. Nat. Nanotechnol. 16, 748–759. 10.1038/s41565-021-00931-2 34211166

[B44] Hong J. Kang M. Jung M. Lee Y. Y. Cho Y. Kim C. (2021). T-cell-derived nanovesicles for cancer immunotherapy. Adv. Mat. 33, 2101110. 10.1002/adma.202101110 34235790

[B45] Hong Y. Nam G. H. Koh E. Jeon S. Kim G. B. Jeong C. (2018). Exosome as a vehicle for delivery of membrane protein therapeutics, PH20, for enhanced tumor penetration and antitumor efficacy. Adv. Funct. Mater. 28, 1703074. 10.1002/adfm.201703074

[B46] Huang X. Wang L. Guo H. Zhang W. (2022). Macrophage membrane-coated nanovesicles for dual-targeted drug delivery to inhibit tumor and induce macrophage polarization. Bioact. Mat. 23, 69–79. 10.1016/j.bioactmat.2022.09.027 PMC9650013 36406251

[B47] Izci M. Maksoudian C. Manshian B. B. Soenen S. J. (2021). The use of alternative strategies for enhanced nanoparticle delivery to solid tumors. Chem. Rev. 121, 1746–1803. 10.1021/acs.chemrev.0c00779 33445874 PMC7883342

[B48] Jang S. C. Kim O. Y. Yoon C. M. Choi D. S. Roh T. Y. Park J. (2013). Bioinspired exosome-mimetic nanovesicles for targeted delivery of chemotherapeutics to malignant tumors. ACS Nano 7, 7698–7710. 10.1021/nn402232g 24004438

[B49] Jia G. Han Y. An Y. Ding Y. He C. Wang X. (2018). NRP-1 targeted and cargo-loaded exosomes facilitate simultaneous imaging and therapy of glioma *in vitro* and *in vivo* . Biomaterials 178, 302–316. 10.1016/j.biomaterials.2018.06.029 29982104

[B50] Jiao Y. Tang Y. Li Y. Liu C. He J. Zhang L. K. (2022). Tumor cell-derived extracellular vesicles for breast cancer specific delivery of therapeutic P53. J. Control. Release 349, 606–616. 10.1016/j.jconrel.2022.07.020 35870568

[B51] Johnstone R. M. (1992). Maturation of reticulocytes: formation of exosomes as a mechanism for shedding membrane proteins. Biochem. Cell. Biol. 70, 179–190. 10.1139/o92-028 1515120

[B52] Kim M. S. Haney M. J. Zhao Y. Yuan D. Deygen I. Klyachko N. L. (2018). Engineering macrophage-derived exosomes for targeted paclitaxel delivery to pulmonary metastases: *in vitro* and *in vivo* evaluations. Nanomedicine 14, 195–204. 10.1016/j.nano.2017.09.011 28982587

[B53] Konoshenko M. Y. Lekchnov E. A. Vlassov A. V. Laktionov P. P. (2018). Isolation of extracellular vesicles: general methodologies and latest trends. Biomed. Res. Int. 2018, 1–27. 10.1155/2018/8545347 PMC5831698 29662902

[B54] Lesterhuis W. J. Haanen J. B. A. G. Punt C. J. A. (2011). Cancer immunotherapy-revisited. Nat. Rev. Drug Discov. 10, 591–600. 10.1038/nrd3500 21804596

[B55] Li G. Jiang Y. Qin Y. Yuan S. Chen X. (2022). Comparing development strategies for PD1/PDL1-based immunotherapies. Nat. Rev. Drug Discov. 21, 484. 10.1038/d41573-022-00003-7 34992259

[B56] Li G. Wang J. Xu M. Zhang H. Tu C. Yang J. (2020). Engineered exosome for NIR-triggered drug delivery and superior synergistic chemo-phototherapy in a glioma model. Appl. Mater Today 20, 100723. 10.1016/j.apmt.2020.100723

[B57] Liang Y. Duan L. Lu J. Xia J. (2021). Engineering exosomes for targeted drug delivery. Theranostics 11, 3183–3195. 10.7150/thno.52570 33537081 PMC7847680

[B58] Lim J. Kostiainen M. Maly J. da Costa V. C. Annunziata O. Pavan G. M. (2013). Synthesis of large dendrimers with the dimensions of small viruses. J. Am. Chem. Soc. 135, 4660–4663. 10.1021/ja400432e 23398590 PMC3638248

[B60] Lin S. Yu Z. Chen D. Wang Z. Miao J. Li Q. (2020). Progress in microfluidics‐based exosome separation and detection technologies for diagnostic applications. Small 16, e1903916. 10.1002/smll.201903916 31663295

[B61] Liu C. Zhang W. Li Y. Chang J. Tian F. Zhao F. (2019). Microfluidic sonication to assemble exosome membrane-coated nanoparticles for immune evasion-mediated targeting. Nano Lett. 19, 7836–7844. 10.1021/acs.nanolett.9b02841 31597431

[B62] Liu H. Li C. Qian Y. Hu L. Fang J. Tong W. (2020). Magnetic-induced graphene quantum dots for imaging-guided photothermal therapy in the second near-infrared window. Biomaterials 232, 119700. 10.1016/j.biomaterials.2019.119700 31881379

[B63] Liu Y. Luo J. Chen X. Liu W. Chen T. (2019). Cell membrane coating technology: a promising strategy for biomedical applications. Nanomicro. Lett. 11, 100. 10.1007/s40820-019-0330-9 34138027 PMC7770915

[B64] Martínez-Greene J. A. Hernández-Ortega K. Quiroz-Baez R. Resendis-Antonio O. Pichardo-Casas I. Sinclair D. A. (2021). Quantitative proteomic analysis of extracellular vesicle subgroups isolated by an optimized method combining polymer‐based precipitation and size exclusion chromatography. J. Extracell. Vesicles 10, e12087. 10.1002/jev2.12087 33936570 PMC8077108

[B65] Matchett E. C. Kornbluth J. (2023). Extracellular vesicles derived from immortalized human natural killer cell line NK3. 3 as a novel therapeutic for multiple myeloma. Front. Immunol. 14, 1265101. 10.3389/fimmu.2023.1265101 37818374 PMC10560732

[B66] McNamara R. P. Caro-Vegas C. P. Costantini L. M. Landis J. T. Griffith J. D. Damania B. A. (2018). Large-scale, cross-flow based isolation of highly pure and endocytosis-competent extracellular vesicles. J. Extracell. Vesicles 7, 1541396. 10.1080/20013078.2018.1541396 30533204 PMC6282418

[B67] Mellman I. Coukos G. Dranoff G. (2011). Cancer immunotherapy comes of age. Nature 480, 480–489. 10.1038/nature10673 22193102 PMC3967235

[B69] Meng Y. Zhang Y. Bühler M. Wang S. Asghari M. Stürchler A. (2023). Direct isolation of small extracellular vesicles from human blood using viscoelastic microfluidics. Sci. Adv. 9, eadi5296. 10.1126/sciadv.adi5296 37801500 PMC10558121

[B70] Mudd A. M. Gu T. Munagala R. Jeyabalan J. Egilmez N. K. Gupta R. C. (2020). Chemoprevention of colorectal cancer by Anthocyanidins and mitigation of metabolic shifts induced by dysbiosis of the gut microbiome. Cancer Prev. Res. 13, 41–52. 10.1158/1940-6207.capr-19-0362 PMC6954290 31796466

[B71] Ngu A. Wang S. Wang H. Khanam A. Zempleni J. (2022). Milk exosomes in nutrition and drug delivery. Am. J. Physiol. Cell. Physiol. 322, C865–C874. 10.1152/ajpcell.00029.2022 35319899 PMC9037700

[B72] Nguyen T. L. Choi Y. Kim J. (2019). Mesoporous silica as A versatile platform for cancer immunotherapy. Adv. Mat. 31, 1803953. 10.1002/adma.201803953 30417454

[B73] Ohno S. Takanashi M. Sudo K. Ueda S. Ishikawa A. Matsuyama N. (2013). Systemically injected exosomes targeted to EGFR deliver antitumor microRNA to breast cancer cells. Mol. Ther. 21, 185–191. 10.1038/mt.2012.180 23032975 PMC3538304

[B74] Onódi Z. Pelyhe C. Terézia Nagy C. Brenner G. B. Laura Almási L. Kittel A. (2018). Isolation of high-purity extracellular vesicles by the combination of iodixanol density gradient ultracentrifugation and bind-elute chromatography from blood plasma. Front. Physiol. 9, 1479. 10.3389/fphys.2018.01479 30405435 PMC6206048

[B75] Park J. Park J. S. Huang C. H. Jo A. Cook K. Wang R. (2021). An integrated magneto-electrochemical device for the rapid profiling of tumour extracellular vesicles from blood plasma. Nat. Biomed. Eng. 5, 678–689. 10.1038/s41551-021-00752-7 34183802 PMC8437135

[B76] Pascucci L. Coccè V. Bonomi A. Ami D. Ceccarelli P. Ciusani E. (2014). Paclitaxel is incorporated by mesenchymal stromal cells and released in Exosomes that inhibit *in vitro* tumor growth: a new approach for drug delivery. J. Control. Release 192, 262–270. 10.1016/j.jconrel.2014.07.042 25084218

[B77] Piffoux M. Nicolás-Boluda A. Mulens-Arias V. Richard S. Rahmi G. Gazeau F. (2019). Extracellular vesicles for personalized medicine: the input of physically triggered production, loading and theranostic properties. Adv. Drug Deliv. Rev. 138, 247–258. 10.1016/j.addr.2018.12.009 30553953

[B78] Piffoux M. Silva A. K. A. Lugagne J. B. Hersen P. Wilhelm C. Gazeau F. (2017). Extracellular vesicle production loaded with nanoparticles and drugs in a trade‐off between loading, yield and purity: towards a personalized drug delivery system. Adv. Biosyst. 1, 1700044. 10.1002/adbi.201700044 32646153

[B79] Poggio M. Hu T. Pai C. C. Chu B. Belair C. D. Chang A. (2019). Suppression of exosomal PD-L1 induces systemic anti-tumor immunity and memory. Cell. 177, 414–427.e13. 10.1016/j.cell.2019.02.016 30951669 PMC6499401

[B80] Pomatto M. A. C. Bussolati B. D’Antico S. Ghiotto S. Tetta C. Brizzi M. F. (2019). Improved loading of plasma-derived extracellular vesicles to encapsulate antitumor miRNAs. Mol. Ther. Methods Clin. Dev. 13, 133–144. 10.1016/j.omtm.2019.01.001 30788382 PMC6370572

[B81] Qi H. Liu C. Long L. Ren Y. Zhang S. Chang X. (2016). Blood exosomes endowed with magnetic and targeting properties for cancer therapy. ACS Nano 10, 3323–3333. 10.1021/acsnano.5b06939 26938862

[B82] Raj S. Khurana S. Choudhari R. Kesari K. K. Kamal M. A. Garg N. (2021). Specific targeting cancer cells with nanoparticles and drug delivery in cancer therapy. Semin. Cancer. Biol. 69, 166–177. 10.1016/j.semcancer.2019.11.002 31715247

[B83] Rao L. Wu L. Liu Z. Tian R. Yu G. Zhou Z. (2020). Hybrid cellular membrane nanovesicles amplify macrophage immune responses against cancer recurrence and metastasis. Nat. Commun. 11, 4909. 10.1038/s41467-020-18626-y 32999291 PMC7527506

[B84] Rayamajhi S. Nguyen T. D. T. Marasini R. Aryal S. (2019). Macrophage-derived exosome-mimetic hybrid vesicles for tumor targeted drug delivery. Acta Biomater. 94, 482–494. 10.1016/j.actbio.2019.05.054 31129363

[B85] Raza F. Zafar H. Zhang S. Kamal Z. Su J. Yuan W. E. (2021). Recent advances in cell membrane-derived biomimetic nanotechnology for cancer immunotherapy. Adv. Healthc. Mat. 10, e2002081. 10.1002/adhm.202002081 33586322

[B86] Ren G. Zhou X. Long R. Xie M. Kankala R. K. Wang S. (2023). Biomedical applications of magnetosomes: state of the art and perspectives. Bioact. Mater 28, 27–49. 10.1016/j.bioactmat.2023.04.025 37223277 PMC10200801

[B87] Ren L. Chen S. Li H. Zhang Z. Zhong J. Liu M. (2016). MRI-guided liposomes for targeted tandem chemotherapy and therapeutic response prediction. Acta Biomater. 15 (35), 260–268. 10.1016/j.actbio.2016.02.011 26873364

[B88] Sahu A. Min K. Jeon J. Yang H. S. Tae G. (2020). Catalytic nanographene oxide with hemin for enhanced photodynamic therapy. J. Control. Release. 326, 442–454. 10.1016/j.jconrel.2020.07.023 32726649

[B89] Salaf T. Zeming K. K. Zhang Y. (2016). Advancements in microfluidics for nanoparticle separation. Lab. Chip 17, 11–33. 10.1039/c6lc01045h 27830852

[B90] Shin S. Jung I. Jung D. Kim C. S. Kang S. M. Ryu S. (2022). Novel antitumor therapeutic strategy using CD4^+^ T cell-derived extracellular Vesicles. Biomaterials 289, 121765. 10.1016/j.biomaterials.2022.121765 36067566

[B91] Siegel R. L. Giaquinto A. N. Jemal A. (2024). Cancer statistics, 2024. CA Cancer J. Clin. 74, 12–49. 10.3322/caac.21820 38230766

[B92] Stolk M. Seifert M. (2015). Protein contaminations impact quantification and functional analysis of extracellular vesicle preparations from mesenchymal stromal cells. J. Stem Cells Regen. Mede. 11, 44–47. 10.46582/jsrm.1102008 PMC4728215 27330254

[B93] Taghikhani A. Hassan Z. M. Ebrahimi M. Moazzeni S. M. (2019). MicroRNA modified tumor-derived exosomes as novel tools for maturation of dendritic cells. J. Cell. Physiol. 234, 9417–9427. 10.1002/jcp.27626 30362582

[B94] Tarach P. Janaszewska A. (2021). Recent advances in preclinical research using PAMAM dendrimers for cancer gene therapy. Int. J. Mol. Sci. 22, 2912. 10.3390/ijms22062912 33805602 PMC7999260

[B95] Tenchov R. Sasso J. M. Wang X. Liaw W. S. Chen C. A. Zhou Q. A. (2022). Exosomes-nature’s lipid nanoparticles, a rising star in drug delivery and diagnostics. ACS Nano 16, 17802–17846. 10.1021/acsnano.2c08774 36354238 PMC9706680

[B96] Tian J. Han Z. Song D. Peng Y. Xiong M. Chen Z. (2023). Engineered exosome for drug delivery: recent development and clinical applications. Int. J. Nanomedicine 18, 7923–7940. 10.2147/IJN.S444582 38152837 PMC10752020

[B97] Tian Y. Li S. Song J. Ji T. Zhu M. Anderson G. J. (2014). A Doxorubicin delivery platform using engineered natural membrane vesicle exosomes for targeted tumor therapy. Biomaterials 35, 2383–2390. 10.1016/j.biomaterials.2013.11.083 24345736

[B98] Tong Z. Yang D. Shen C. Li C. Xu X. Li Q. (2024). Rapid automated extracellular vesicle isolation and miRNA preparation on a cost-effective digital microfluidic platform. Anal. Chim. Acta 1296, 342337. 10.1016/j.aca.2024.342337 38401929

[B99] Upadhaya S. Neftelino S. T. Hodge J. P. Oliva C. Campbell J. R. Yu J. X. (2021). Combinations take centre stage in PD1/PDL1 inhibitor clinical trials. Nat. Rev. Drug Discov. 20, 168–169. 10.1038/d41573-020-00204-y 33177720

[B100] Wang J. Li G. Tu C. Chen X. Yang B. Huo Y. (2020). High-throughput single-cell analysis of exosome mediated dual drug delivery, *in vivo* fate and synergistic tumor therapy. Nanoscale 12, 13742–13756. 10.1039/d0nr02344b 32573602

[B101] Wang Q. Li T. Yang J. Zhao Z. Tan K. Tang S. (2022). Engineered exosomes with independent module/cascading function for therapy of Parkinson's disease by multistep targeting and multistage intervention method. Adv. Mat. 34, 2201406. 10.1002/adma.202201406 35435282

[B102] Welsh J. A. Goberdhan D. C. I. O'Driscoll L. Buzas E. I. Blenkiron C. Bussolati B. (2024). Minimal information for studies of extracellular vesicles (MISEV2023): from basic to advanced approaches. J. Extracell. Vesicles 13, e12404. 10.1002/jev2.12404 38326288 PMC10850029

[B103] Wen Y. Fu Q. Soliwoda A. Zhang S. Zheng M. Mao W. (2022). Cell-derived nanovesicles prepared by membrane extrusion are good substitutes for natural extracellular vesicles. Extracell. vesicle 1, 100004. 10.1016/j.vesic.2022.100004 36578271 PMC9794200

[B104] Wilhelm S. Tavares A. J. Dai Q. Ohta S. Audet J. Dvorak H. F. (2016). Analysis of nanoparticle delivery to tumours. Nat. Rev. Mat. 1, 16014. 10.1038/natrevmats.2016.14

[B105] Xiang X. Guan F. Jiao F. Li H. Zhang W. Zhang Y. (2021). A new urinary exosome enrichment method by a combination of ultrafiltration and TiO 2 nanoparticles. Anal. Methods 13, 1591–1600. 10.1039/d1ay00102g 33729255

[B106] Xiao T. Ma Y. Zhang Z. Zhang Y. Zhao Y. Zhou X. (2024). Tailoring therapeutics via a systematic beneficial elements comparison between photosynthetic bacteria-derived OMVs and extruded nanovesicles. Bioact. Mat. 36, 48–61. 10.1016/j.bioactmat.2024.02.025 PMC10904884 38434148

[B108] Xie J. Haesebrouck F. Hoecke L. Vandenbroucke R. E. (2023). Bacterial extracellular vesicles: an emerging avenue to tackle diseases. Trends Microbiol. 31, 1206–1224. 10.1016/j.tim.2023.05.010 37330381

[B109] Xie M. Wu D. Li G. Yang J. Zhang Y. S. (2020). Exosomes targeted towards applications in regenerative medicine. Nano Sel. 2, 880–908. 10.1002/nano.202000251

[B110] Xu R. Fitts A. Li X. Fernandes J. Pochampally R. Mao J. (2016). Quantification of small extracellular vesicles by size exclusion chromatography with fluorescence detection. Anal. Chem. 88, 10390–10394. 10.1021/acs.analchem.6b03348 27689436 PMC5089915

[B111] Xu T. Fan L. Wang L. Ren H. Zhang Q. Sun W. (2024). Hierarchical mesoporous silicon and albumin composite microparticles delivering Dox and FU for liver cancer treatment. Int. J. Biol. Macromol. 268, 131732. 10.1016/j.ijbiomac.2024.131732 38649078

[B112] Xu Y. Feng K. Zhao H. Di L. Wang L. Wang R. (2022). Tumor-derived extracellular vesicles as messengers of natural products in cancer treatment. Theranostics 12, 1683–1714. 10.7150/thno.67775 35198064 PMC8825588

[B113] Yan G. Xiao Q. Zhao J. Chen H. Xu Y. Tan M. (2024). Brucea javanica derived exosome-like nanovesicles deliver miRNAs for cancer therapy. J. Control. Release 367, 425–440. 10.1016/j.jconrel.2024.01.060 38295998

[B114] Yang D. Zhang W. Zhang H. Zhang F. Chen L. Ma L. (2020). Progress, opportunity, and perspective on exosome isolation-efforts for efficient exosome-based theranostics. Theranostics 10, 3684–3707. 10.7150/thno.41580 32206116 PMC7069071

[B115] Yang F. Liao X. Tian Y. Li G. (2017). Exosome separation using microfluidic systems: size‐based, immunoaffinity‐based and dynamic methodologies. Biotechnol. J. 12, 4. 10.1002/biot.201600699 28166394

[B116] Yin Z. Yu M. Ma T. Zhang C. Huang S. Karimzadeh M. R. (2021). Mechanisms underlying low-clinical responses to PD-1/PD-L1 blocking antibodies in immunotherapy of cancer: a key role of exosomal PD-L1. J. Immunother. Cancer 9, e001698. 10.1136/jitc-2020-001698 33472857 PMC7818841

[B117] Yong T. Wang D. Li X. Yan Y. Hu J. Gan L. (2020). Extracellular vesicles for tumor targeting delivery based on five features principle. J. Control. Release 322, 555–565. 10.1016/j.jconrel.2020.03.039 32246977

[B118] Yong T. Wei Z. Gan L. Yang X. (2022). Extracellular-vesicle-based drug delivery systems for enhanced antitumor therapies through modulating the cancer-immunity cycle. Adv. Mat. 34, e2201054. 10.1002/adma.202201054 35726204

[B119] Yong T. Zhang X. Bie N. Zhang H. Zhang X. Li F. (2019). Tumor exosome-based nanoparticles are efficient drug carriers for chemotherapy. Nat. Commun. 10, 3838. 10.1038/s41467-019-11718-4 31444335 PMC6707218

[B120] You J. Y. Kang S. J. Rhee W. J. (2021). Isolation of cabbage exosome-like nanovesicles and investigation of their biological activities in human cells. Bioact. Mat. 6, 4321–4332. 10.1016/j.bioactmat.2021.04.023 PMC8105599 33997509

[B121] Zhang H. Li G. Yang J. Chen A. Xie M. (2021). Supercritical-derived artemisinin microfibers and microparticles for improving anticancer effects. J. Supercrit. Fluid 175, 105276. 10.1016/j.supflu.2021.105276

[B122] Zhang J. Wan S. Zhou H. Du J. Li Y. Zhu H. (2024). Programmed nanocloak of commensal bacteria-derived nanovesicles amplify strong immunoreactivity against tumor growth and metastatic progression. ACS Nano 18, 9613–9626. 10.1021/acsnano.3c13194 38502546

[B123] Zhang K. L. Wang Y. J. Sun J. Zhou J. Xing C. Huang G. (2019). Artificial chimeric exosomes for anti-phagocytosis and targeted Cancer therapy. Chem. Sci. 10, 1555–1561. 10.1039/c8sc03224f 30809374 PMC6357862

[B124] Zhang M. Jin K. Gao L. Zhang Z. Li F. Zhou F. (2018). Methods and technologies for exosome isolation and characterization. Small Methods 2, 1800021. 10.1002/smtd.201800021

[B125] Zhang M. Xiao B. Wang H. Han M. K. Zhang Z. Viennois E. (2016). Edible ginger-derived nano-lipids loaded with Doxorubicin as a novel drug-delivery approach for colon cancer therapy. Mol. Ther. 24, 1783–1796. 10.1038/mt.2016.159 27491931 PMC5112046

[B126] Zhang W. Yu Z. L. Wu M. Ren J. G. Xia H. F. Sa G. L. (2017). Magnetic and folate functionalization enables rapid isolation and enhanced tumor-targeting of cell-derived microvesicles. ACS Nano 11, 277–290. 10.1021/acsnano.6b05630 28005331

[B127] Zhang X. Zhang H. Gu J. Zhang J. Shi H. Qian H. (2021). Engineered extracellular vesicles for cancer therapy. Adv. Mat. 33, 2005709. 10.1002/adma.202005709 33644908

[B128] Zhao M. Cheng X. Shao P. Dong Y. Wu Y. Xiao L. (2024). Bacterial protoplast-derived nanovesicles carrying CRISPR-Cas9 tools re-educate tumor-associated macrophages for enhanced cancer immunotherapy. Nat. Commun. 15, 950. 10.1038/s41467-024-44941-9 38296939 PMC10830495

[B129] Zhao Q. Wang T. Wang H. Cao P. Jiang C. Qiao H. (2023). Consensus statement on research and application of Chinese herbal medicine derived extracellular vesicles-like particles (2023 edition). Chin. Herb. Med. 16, 3–12. 10.1016/j.chmed.2023.11.002 38375050 PMC10874762

[B130] Zhong Y. Wang X. Zhao X. Shen J. Wu X. Gao P. (2023). Multifunctional milk-derived small extracellular vesicles and their biomedical applications. Pharmaceutics 15, 1418. 10.3390/pharmaceutics15051418 37242660 PMC10223436

[B131] Zhu L. Sun H. T. Wang S. Huang S. L. Zheng Y. Wang C. Q. (2020). Isolation and characterization of exosomes for cancer research. J. Hematol. Oncol. 13, 152. 10.1186/s13045-020-00987-y 33168028 PMC7652679

[B132] Zhu Z. Zhai Y. Hao Y. Wang Q. Han F. Zheng W. (2022). Specific anti‐glioma targeted‐delivery strategy of engineered small extracellular vesicles dual‐functionalised by Angiopep‐2 and TAT peptides. J. Extracell.Vesicles 11, e12255. 10.1002/jev2.12255 35932288 PMC9451528

[B133] Zhuang M. Chen X. Du D. Shi J. Deng M. Long Q. (2020). SPION decorated exosome delivery of TNF-α to cancer cell membranes through magnetism. Nanoscale 12, 173–188. 10.1039/c9nr05865f 31803890

[B134] Zhuang X. Teng Y. Samykutty A. Mu J. Deng Z. Zhang L. (2016). Grapefruit-derived nanovectors delivering therapeutic miR17through an intranasal route inhibit brain tumor progression. Mol. Ther. 24, 96–105. 10.1038/mt.2015.188 26444082 PMC4754550

[B135] Zhuo Z. Wang J. Luo Y. Zeng R. Zhang C. Zhou W. (2021). Targeted extracellular vesicle delivery systems employing superparamagnetic iron oxide nanoparticles. Acta biomater. 134, 13–31. 10.1016/j.actbio.2021.07.027 34284151

[B136] Zu M. Xie D. Canup B. S. B. Chen N. Wang Y. Sun R. (2021). Green’ nanotherapeutics from tea leaves for orally targeted prevention and alleviation of colon diseases. Biomaterials 279, 121178. 10.1016/j.biomaterials.2021.121178 34656857

